# Krüppel-like factor 8 activates the transcription of C-X-C cytokine receptor type 4 to promote breast cancer cell invasion, transendothelial migration and metastasis

**DOI:** 10.18632/oncotarget.8083

**Published:** 2016-03-14

**Authors:** Debarati Mukherjee, Heng Lu, Lin Yu, Chunjiang He, Satadru K. Lahiri, Tianshu Li, Jihe Zhao

**Affiliations:** ^1^ Burnett School of Biomedical Sciences, University of Central Florida College of Medicine, Orlando, FL, USA; ^2^ School of Basic Medical Sciences, Wuhan University, Wuhan, China; ^3^ Current address: Cleveland Clinic, Cleveland, OH, USA

**Keywords:** KLF8, CXCR4, transendothelial migration, invasion, metastasis

## Abstract

Krüppel-like factor 8 (KLF8) has been strongly implicated in breast cancer metastasis. However, the underlying mechanisms remain largely unknown. Here we report a novel signaling from KLF8 to C-X-C cytokine receptor type 4 (CXCR4) in breast cancer. Overexpression of KLF8 in MCF-10A cells induced CXCR4 expression at both mRNA and protein levels, as determined by quantitative real-time PCR and immunoblotting. This induction was well correlated with increased Boyden chamber migration, matrigel invasion and transendothelial migration (TEM) of the cells towards the ligand CXCL12. On the other hand, knockdown of KLF8 in MDA-MB-231 cells reduced CXCR4 expression associated with decreased cell migration, invasion and TEM towards CXCL12. Histological and database mining analyses of independent cohorts of patient tissue microarrays revealed a correlation of aberrant co-elevation of KLF8 and CXCR4 with metastatic potential. Promoter analysis indicated that KLF8 directly binds and activates the human CXCR4 gene promoter. Interestingly, a CXCR4-dependent activation of focal adhesion kinase (FAK), a known upregulator of KLF8, was highly induced by CXCL12 treatment in KLF8-overexpressing, but not KLF8 deficient cells. This activation of FAK in turn induced a further increase in KLF8 expression. Xenograft studies showed that overexpression of CXCR4, but not a dominant-negative mutant of it, in the MDA-MB-231 cells prevented the invasive growth of primary tumor and lung metastasis from inhibition by knockdown of KLF8. These results collectively suggest a critical role for a previously unidentified feed-forward signaling wheel made of KLF8, CXCR4 and FAK in promoting breast cancer metastasis and shed new light on potentially more effective anti-cancer strategies.

## INTRODUCTION

Breast cancer is one of the leading causes of cancer-related deaths among women [1, 2]. Current mainstream strategies including surgical resection and adjuvant therapies are effective for well-confined primary tumors only. However, metastatic breast cancer remains largely incurable and responsible for 90% of the patient deaths [3, 4]. Metastasis is an event of sequential steps including initial dissociation of some cells from the primary tumor, invasion through the stroma to access vascular vessels, penetration through the vessel wall via TEM to enter the blood or intravasation, traveling with blood, leaving the circulation via TEM once again or extravasation to land on distant organs/tissues, initial secondary growth to form a dormant micrometastasis, and finally re-initiation of a massive growth to form metastasis or colonization [5]. Yet, the precise mechanisms underlying these steps remain poorly understood. A proper elucidation of these mechanisms is critical for better diagnostic and therapeutic strategies.

KLF8 is a dual transcription factor known to express at marginally detectable levels in most normal tissue types and aberrantly overexpress in a number of human cancer types including breast cancer [6]. Cell culture studies have demonstrated a critical role of KLF8 in promoting cell cycle progression [6–8], transformation [9, 10], epithelial-to-mesenchymal transition (EMT) [6, 11–13], cancer stem cell induction [14] and DNA-damage response [15]. Patient tissue analyses have revealed a strong correlation of KLF8 expression with metastatic potential [15, 16]. Xenograft studies have pointed to the role of KLF8 in promoting invasive growth and metastasis of breast cancer [16–19]. Despite the discovery of an array of target genes and microRNAs of KLF8 that are associated with cancer [6, 9, 14] and mechanisms regulating KLF8 at protein and subcellular levels [6, 15, 20–25], research on the molecular mechanisms by which KLF8 promotes metastasis remains in its infancy.

CXCR4 is a receptor for chemokine CXCL12 and has recently been linked to breast cancer metastasis [26–28]. The receptor-ligand interaction was initially implicated in HIV infection of T lymphocytes [29–32], chemotaxis and homing of CXCR4-high leucocytes and hematopoietic stem cells to CXCL12-rich inflammatory sites, lymphoid organs and bone marrow [26]. Recent studies have drawn much attention to CXCR4-high cancer cell metastasis to CXCL12-rich tissues suggesting that such cancer cells have hijacked the CXCL12- guided mechanism of cell homing to establish metastasis [26, 27, 33–36]. Indeed, breast cancer cells metastasize preferentially to CXCL12-rich tissues such as the lungs and bone marrow [3, 26, 27, 33–35, 37]. Inhibition of CXCR4-CXCL12 interaction has led to reduction in experimental metastasis of breast cancer [38–40]. Aberrant high levels of CXCR4 have been found in patient tissues of breast cancer [34, 35, 41, 42].

In this report, we show strong evidence that KLF8 directly activates the CXCR4 gene promoter. This activation causes an increased CXCR4 expression and subsequent feed-forward activation of FAK, resulting in CXCR4/CXCL12-dependent increase in migration, invasion, TEM and metastasis of breast cancer cells. These novel findings may guide development of more effective and less toxic, targeted therapeutic strategies.

## RESULTS

### KLF8 upregulates CXCR4 expression in invasive breast cancer

**Figure 1:**
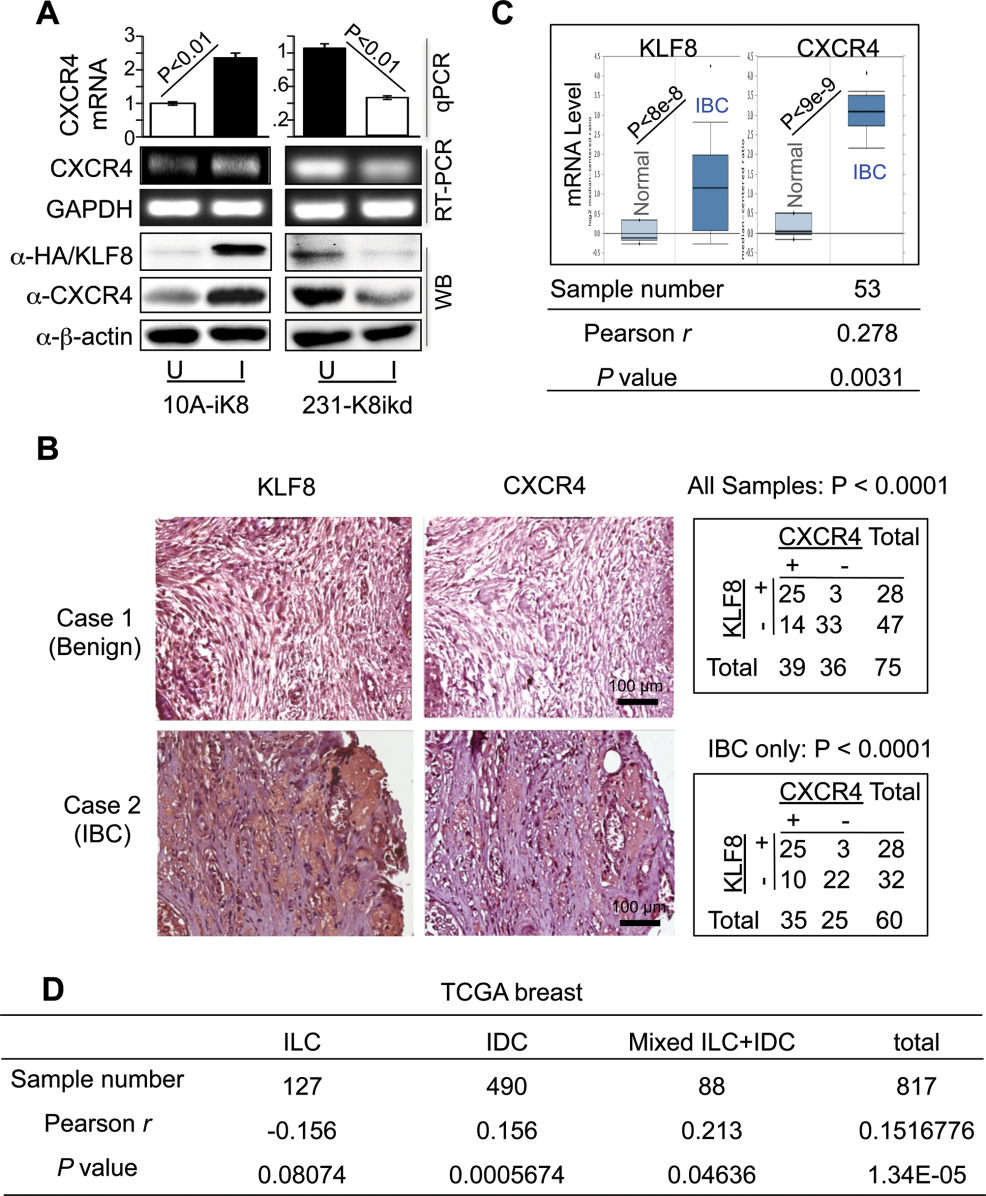
KLF8 upregulates CXCR4 expression associated with invasive potential (**A**) Overexpression and knockdown of KLF8 induces and reduces CXCR4 expression in the 10A-iK8 and 231-K8ikd cells, respectively. Total RNA and protein lyaste were prepared from sub-confluent cells grown under uninduced (U) and doxycycline-induced (I) conditions. The levels of CXCR4 were determined by qRT–PCR (top graph), semi-quantitative RT–PCR (middle panel) or western blotting (bottom panel). Overexpression of HA-KLF8 and knockdown of KLF8 were examined by western blot. (**B**) Aberrant co-elevation of KLF8 and CXCR4 protein in the patient invasive breast carcinoma (IBC) tumors. IHC staining was performed for KLF8 or CXCR4 (brown) in the human breast cancer tissue microarray containing specimens in duplicate from 75 patient tumors or normal tissues. Images representing a benign sample negative for both KLF8 and CXCR4 (case 1) and an IBC sample positive for both KLF8 and CXCR4 (case 2) are shown. Correlation of CXCR4 and KLF8 expression is outlined in the tables. (**C**) Aberrant co-elevation of KLF8 and CXCR4 mRNA in the patient IBC tumors. Oncomine analysis was performed on an independent cohort of Finak dataset containing 6 normal and 53 IBC samples. Pearson's correlation of KLF8 and CXCR4 was applied to Finak dataset downloaded from GEO (GSE9014). (**D**) The expression of KLF8 and CXCR4 is correlated in invasive breast cancers. RNA sequencing data downloaded from TCGA breast cancer (2015) were analyzed.

Aberrant CXCR4 overexpression has been implicated in breast tumor growth and metastasis [27, 34, 41, 43]. Our previous study showed that KLF8 expression is undetectable in breast epithelial cells like MCF-10A, while it is aberrantly overexpressed in invasive breast cancer cells such as MDA-MB-231 [11]. Furthermore, CXCR4 is highly expressed in MDA-MB-231 cell line and shows very low expression in MCF-10A cells. Our previous cDNA microarray analysis showed that CXCR4 mRNA expression is significantly upregulated upon overexpression of KLF8 [44]. To verify this result, we examined the changes in CXCR4 expression in response to doxycycline-induced overexpression or knockdown of KLF8 in our MCF-10A cell line expressing inducible KLF8 (or 10A-iK8) and MDA-MB-231 cell line expressing inducible shRNA against KLF8, respectively [16–19] using quantitative real-time PCR (qRT-PCR) (Figure 1A, top panel), semi-quantitative reverse-transcription PCR (Figure 1A, middle panel), and western blotting (Figure 1A, bottom panel). The expression of CXCR4 was markedly increased at both mRNA and protein levels when KLF8 expression was induced in the CXCR4-low 10A-iK8. Conversely, knockdown of KLF8 in the CXCR4-high 231-K8ikd cells [45–48] led to significant CXCR4 reduction.

It is known that both KLF8 and CXCR4 are aberrantly overexpressed in breast cancer [26, 27, 36]. To test if KLF8 and CXCR4 are co-overexpressed in patient tumors, we performed immunohistochemical (IHC) staining for CXCR4 in a human breast cancer tissue microarray in which KLF8 expression was determined previously [16–19] (Figure 1B, images). We found that 89.28% of the KLF8-positive tumors also express CXCR4. By contrast, among the KLF8-negative samples, only 29.78% express CXCR4 (Figure 1B, top-right table). We also found that their co-expression is positively correlated with the invasive potential (Figure 1B, bottom-right table). This result was further demonstrated by Oncomine analysis of an independent cohort of patient tissues (Figure 1C top). Furthermore, microarray data profiling of invasive human breast cancer tissues downloaded from GEO and TCGA database was applied to examine the co-expression of KLF8 and CXCR4. The Pearson's correlation analysis identified the positive correlation of KLF8 and CXCR4 in invasive breast cancers (Figure 1C bottom and Figure 1D), specifically in invasive ductal carcinoma (IDC) rather than invasive lobular carcinoma (ILC) (Figure 1D).

These results strongly suggest that CXCR4 expression could be aberrantly upregulated by KLF8 in breast cancer cells particularly in invasive tumors.

### KLF8 directly targets CXCR4 gene promoter for transcriptional activation

**Figure 2:**
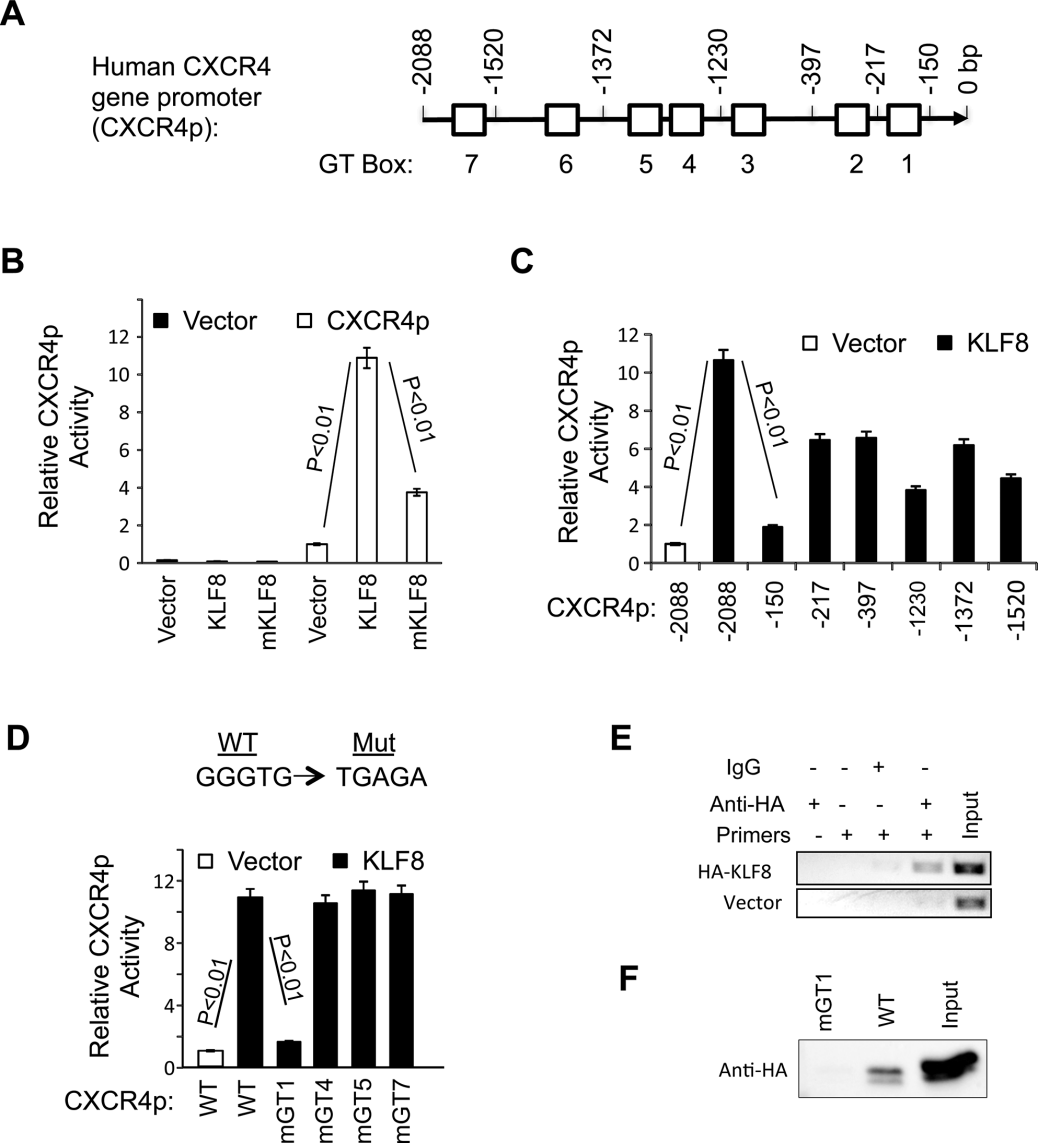
KLF8 upregulates CXCR4 at the level of transcription (**A**) Schematic diagram of CXCR4p. Potential KLF8-binding sites (GT boxes) are shown. The CXCR4p was isolated from MDA-MB-231 genomic DNA and inserted in the pGL3basic luciferase reporter vector. (**B**) KLF8 activates the CXCR4p. The CXCR4p or control vector was co-transfected with KLF8 or activation domain-defective mutant (mKLF8) into NIH3T3 cells. Reporter activity was performed as described in Materials and Methods. (**C**) KLF8 responsive site is located between −150 and −217 bp of the CXCR4p. Serial CXCR4 truncation mutants were constructed and tested for changes in the promoter activation by KLF8. (**D**) The GT-box 1 is required for KLF8 to activate the CXCR4p. Indicated GT boxes were mutated (mGT) and tested for changes in the promoter activation by KLF8. (**E**, **F**) KLF8 directly binds CXCR4p at the GT-box 1 site. HA-KLF8 was overexpressed in HEK293 cells. The cells were processed for either ChIP assay using primers spanning the GT-box 1 (E) or BOP assay using oligos spanning the wild type GT1 (WT) or its mutant (mGT1) (F) as described in Materials and Methods.

KLF8 regulates target gene promoters by binding to a CACCC (or GGGTG) or GT-box site [21, 49]. Since our results indicated that KLF8 can induce CXCR4 expression at both mRNA and protein levels, we tested whether KLF8 regulates the transcription of CXCR4. We cloned the human CXCR4 gene promoter (CXCR4p) that contains seven GT-boxes (Figure 2A). We first tested if the activity of the CXCR4p can be regulated by KLF8. The promoter reporter assay showed that co-expression of KLF8 in NIH3T3 (as well as HEK293. Not shown) cells caused > 10-fold increase in the promoter activity (Figure 2B). By contrast, the activation domain-deficient mutant of KLF8 (mKLF8) [21] failed to do so. This result suggests that KLF8 regulates CXCR4 expression by transcriptional activation.

We then attempted to map the KLF8 responding region in CXCR4p by step-wise truncation of CXCR4p. We found that truncation through −217 bp position did not significantly prevent the promoter from responding to KLF8 until the deletion to the −150 bp position (Figure 2C). This result suggests that the promoter region between −217 bp and −150 bp is the most critical for the activation of CXCR4p by KLF8. And this region contains GT-box 1.

We then tried to determine if the GT-box 1 plays a role for the activation of CXCR4p by KLF8 by disabling the GT-box 1 in the full-length CXCR4p. Subsequent reporter assays showed that activation of CXCR4p by KLF8 was completely prevented by mutation of the GT-box 1, but none of the other GT-boxes indicated (Figure 2D). This result suggests that the GT-box 1 site is required for KLF8-mediated activation of CXCR4p.

To determine if KLF8 directly binds GT-box 1, we performed chromatin immunoprecipitation (ChIP) assays using HEK293 cells overexpressing HA-KLF8. A fragment of endogenous CXCR4p that spans the GT-box 1 was specifically co-precipitated by the anti-HA antibody but not by the control IgG (Figure 2E). This was further corroborated by biotinylated-oligonucleotide precipitation (BOP) assay showing that KLF8 can bind the CXCR4 promoter region in a GT-box 1-dependent manner given that the binding was abolished by GT-box 1 disruption (mGT1) (Figure 2F).

These results demonstrate that KLF8 directly activates the transcription of CXCR4 likely through binding CXCR4p at GT-box 1.

### KLF8 promotes CXCL12-stimulated cell migration and invasion

**Figure 3:**
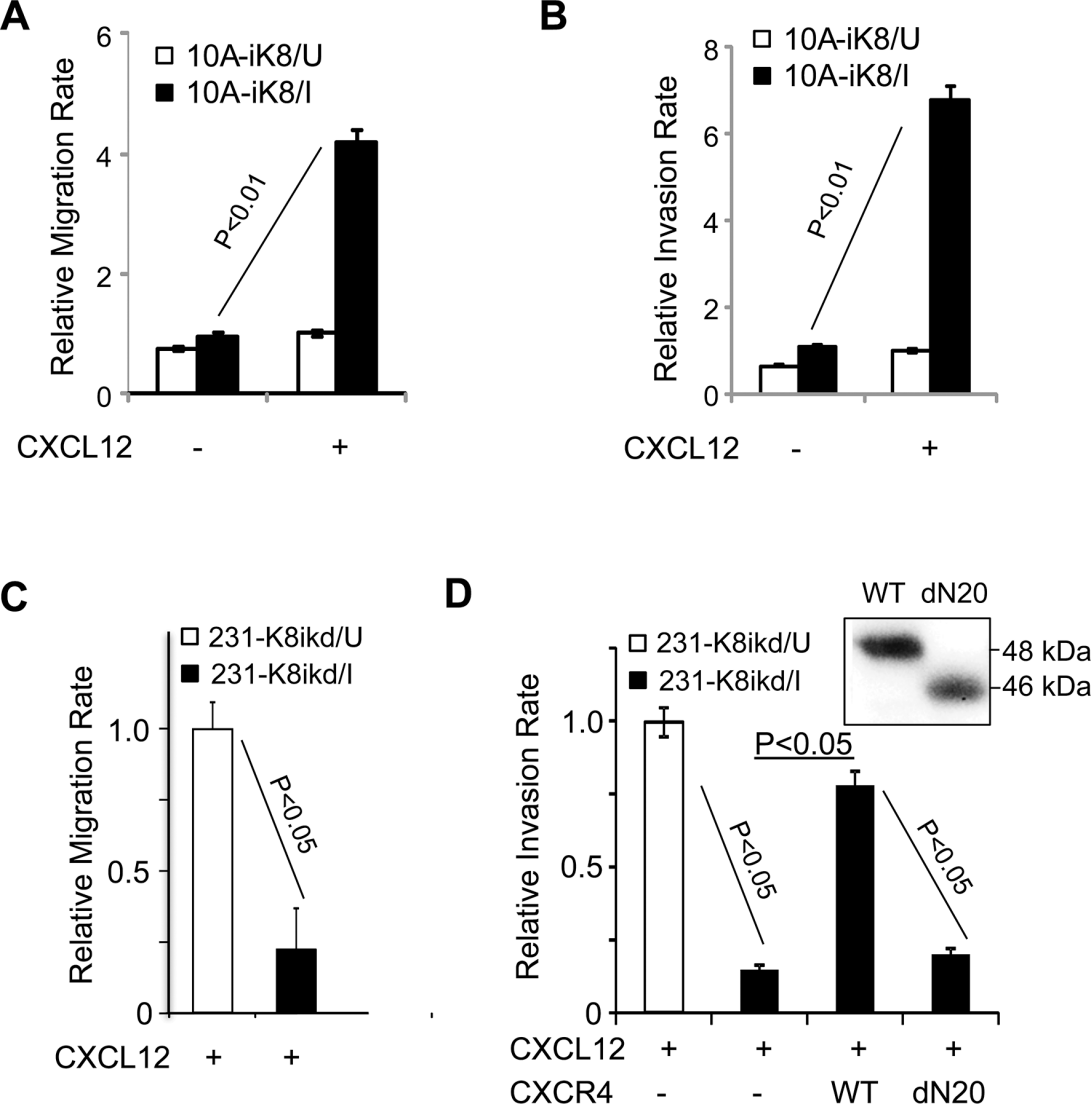
KLF8 promotes cell migration and invasion towards CXCL12 (**A**, **B**) Overexpression of KLF8 increases migration and invasion towards CXCL12. The 10A-ik8 cells grown under U or I conditions were subject to Boyden chamber migration Assay (a) or Matrigel invasion assay (b). (**C**, **D**) Knockdown of KLF8 inhibits CXCR4-dependent migration and invasion towards CXCL12. The 231-K8ikd cells grown under U or I conditions with or without overexpression of CXCR4 (WT) or its ligand-binding defective mutant (dN20) were subject to Boyden chamber migration Assay (c) or Matrigel invasion assay (d). No detectable migration or invasion was observed towards serum-free medium in the absence of CXCL12. Inset, ectopic expression of CXCR4 and the dN20 mutant were verified by anti-HA western blotting.

CXCR4 mediates cell trafficking along a chemotactic gradient CXCL12 [26, 27, 36]. To test if KLF8 can positively regulate cell migration and invasion towards CXCL12, we performed Boyden chamber migration and matrigel invasion assays, respectively. The rates of both the cell migration and invasion were significantly increased when overexpression of KLF8 was induced in the 10A-iK8 cells (Figure 3A and 3B). Conversely, the 231K8-ikd cells migrated and invaded at a much slower rate upon knockdown of KLF8 (Figure 3C and 3D). In the absence of CXCL12 as a chemoattractant, migration and invasion rate of 231K8-ikd cells remains unchanged upon KLF8 knockdown [16] (data not shown). Importantly, the knockdown-induced decrease in invasion was fully recovered by stable overexpression of CXCR4, but not its ligand binding-defective mutant dN20 [50] (Figure 3D).

These results strongly suggest that KLF8 promotes CXCL12-dependant migration and invasion by upregulating the expression of CXCR4 in the cell.

### KLF8 promotes CXCR4-dependent transendothelial migration (TEM) towards CXCL12

**Figure 4:**
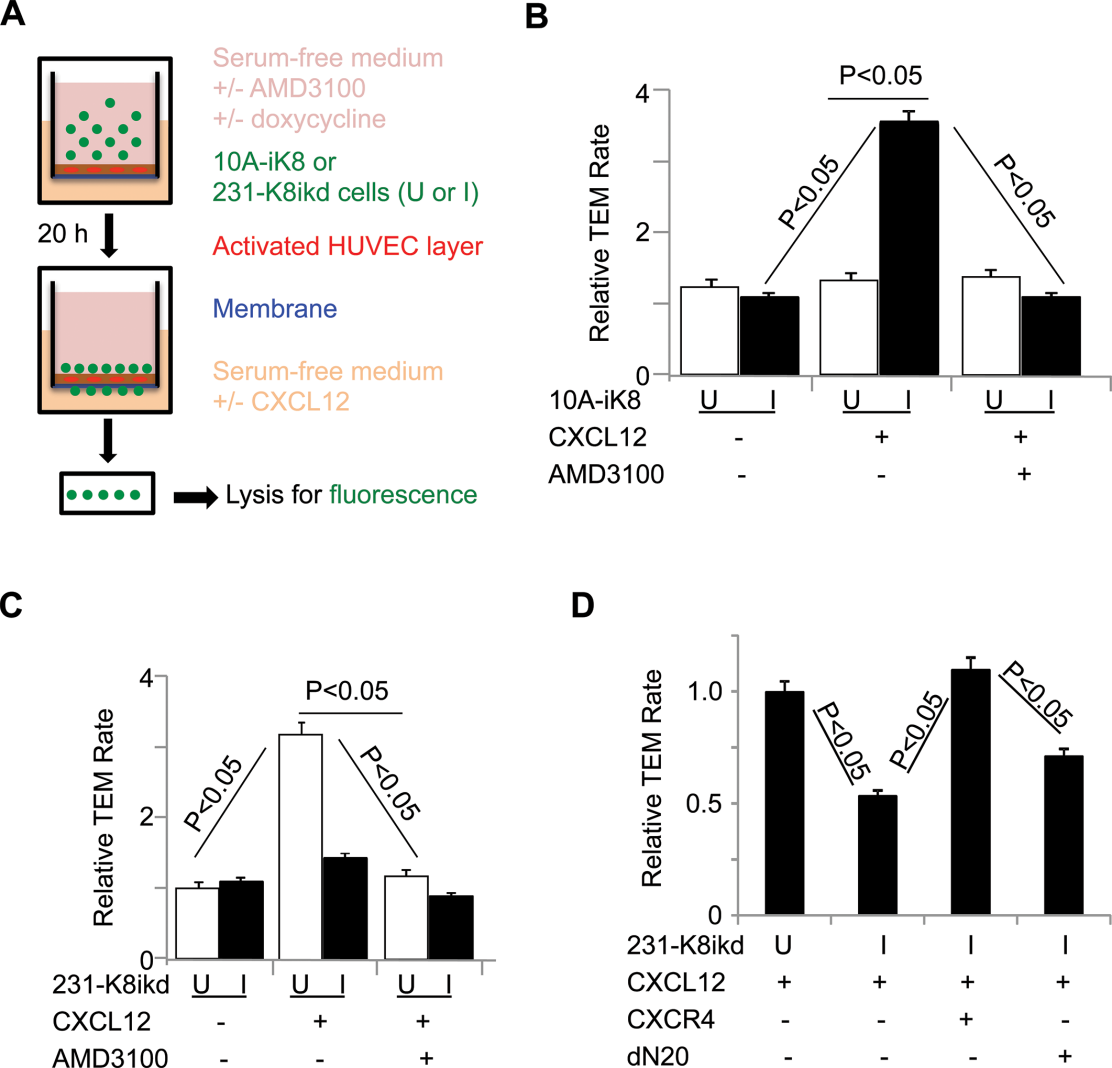
KLF8 promotes CXCR4-dependent TEM towards CXCL12 (**A**) Illustration of the procedure for the TEM assay. (**B**) Overexpression of KLF8 is sufficient to promote CXCR4-dependent TEM of 10A-iK8 cells towards CXCL12. (**C**) Knockdown of KLF8 inhibits CXCR4-dependent TEM of 231-K8ikd cells towards CXCL12. (**D**) Overexpression of CXCR4 but not the dN20 mutant prevents TEM of 231-K8ikd cells towards CXCL12 from inhibition by knockdown of KLF8.

Metastatic tumor cell disseminates by invading the stromal matrix, followed by crossing the endothelial barrier via TEM to enter the blood stream. TEM is further implicated in the exit of circulating tumor cells from blood stream to land on a distant site [51]. Previous reports suggested that the CXCR4/CXCL12 signaling-mediated chemotaxis may be involved in migration of CXCR4^+^ cancer cells towards an increasing gradient of CXCL12 across vascular wall [51, 52]. We thus tested if KLF8 promotes CXCR4-dependent TEM towards CXCL12 (Figure 4A). We found that induction of KLF8 overexpression in the 10A-iK8 cells led to a > 2-fold increase in TEM rate which was blocked by the CXCR4-specific antagonist AMD3100 [36] (Figure 4B). Conversely, TEM rate of the 231-K8ikd cells was markedly reduced upon knockdown of KLF8 or treatment with AMD3100 (Figure 4C). Overexpression of CXCR4, but not its dN20 mutant, protected the TEM capability of the cells from inhibition by knockdown of KLF8 (Figure 4D). These results strongly indicate that KLF8 plays a critical role in promoting TEM requiring CXCR4 engagement during intravasation and/or extravasation.

### Upregulation of CXCR4 by KLF8 leads to a CXCL12-dependent feed-forward activation of FAK upstream of KLF8

**Figure 5:**
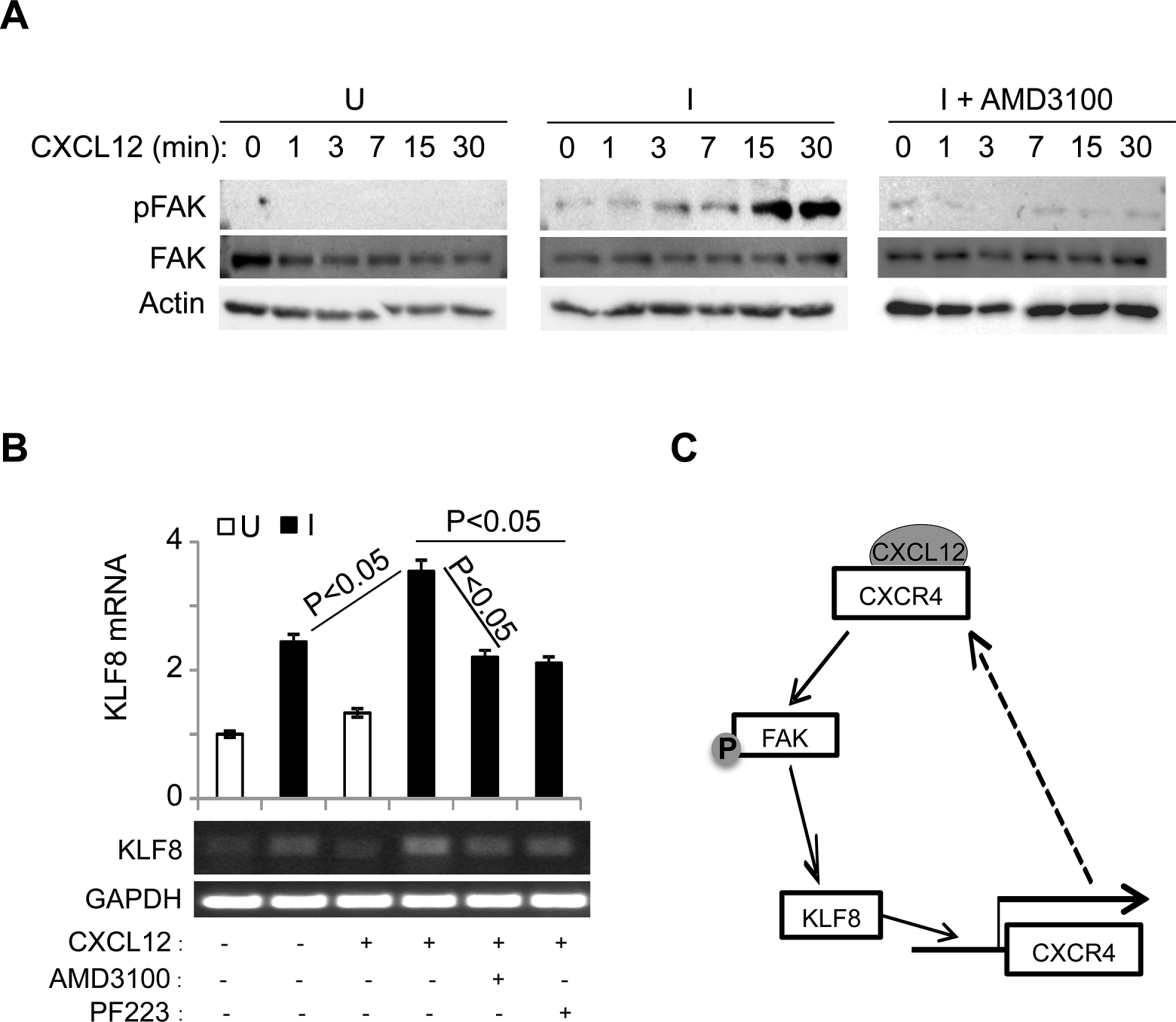
The upregulation of CXCR4 by KLF8 leads to a feed-forward activation of FAK upstream of KLF8 (**A**) Overexpression of KLF8 causes CXCL12/CXCR4-dependent activation of FAK. Uninduced (U) and induced (I) 10A-iK8 cells were serum-starved for 24 hours followed by 3-hour treatment with AMD3100 (35 ng/ml) or mock treatment prior to CXCL12 (100 ng/ml) stimulation. Whole cell lysates were prepared for western blotting for FAK phosphorylation at Y397 (pFAK) and total FAK. (**B**) Overexpression of KLF8 induces CXCL12/CXCR4-dependent expression of endogenous KLF8. The 10A-iK8 cells were grown and treated similarly as in A except for inclusion of PF223, a FAK-specific inhibitor. Total RNA was prepared for qRT-PCR (top panel) and semi-quantitative RT-PCT (bottom panel). (**C**) Hypothetic model for the feed-forward signaling loop of KLF8 to CXCR4/CXCL12 to pFAK and back to KLF8.

Previous reports suggested that engagement of CXCR4 by CXCL12 causes the activation of FAK that contributes to CXCL12-dependant chemotaxis in breast cancer cells [53, 54]. To test if FAK can be activated by CXCL12 stimulation and whether KLF8 plays a role, we performed western blotting for the phosphorylation of FAK at tyrosine 397 (or pFAK) [55, 56] in the 10A- iK8 cells (Figure 5A). When the overexpression of KLF8 was not induced, CXCL12 treatment could not activate FAK (Figure 5A, left panel). By contrast, when the overexpression of KLF8 was induced, CXCL12 stimulated a rapid activation of FAK without affecting the overall expression of FAK (Figure 5A, middle panel), which was prevented by treatment with AMD3100 (Figure 5A, right panel). These results suggest that the induction of CXCR4 expression by KLF8 and subsequent ligand engagement lead to the activation of FAK.

Interestingly, FAK was initially identified as the upstream inducer of KLF8 expression [8]. We thus tested if the CXCL12-stimulated FAK activation increases KLF8 expression. We found that CXCL12 exposure led to a further increase in KLF8 mRNA level in the 10A-iK8 cells when the overexpression of the ectopic KLF8 was induced, which was prevented by pre-inactivation of either CXCR4 or FAK (Figure 5B).

Taken together, these results point to a potentially important feed-forward signaling wheel consisting of KLF8, CXCR4 and FAK and CXCL12 is a critical driving force for the cycling of the wheel in breast cancer cells (Figure 5C).

### KLF8 promotes CXCR4-dependent invasive growth of the primary tumor

**Figure 6:**
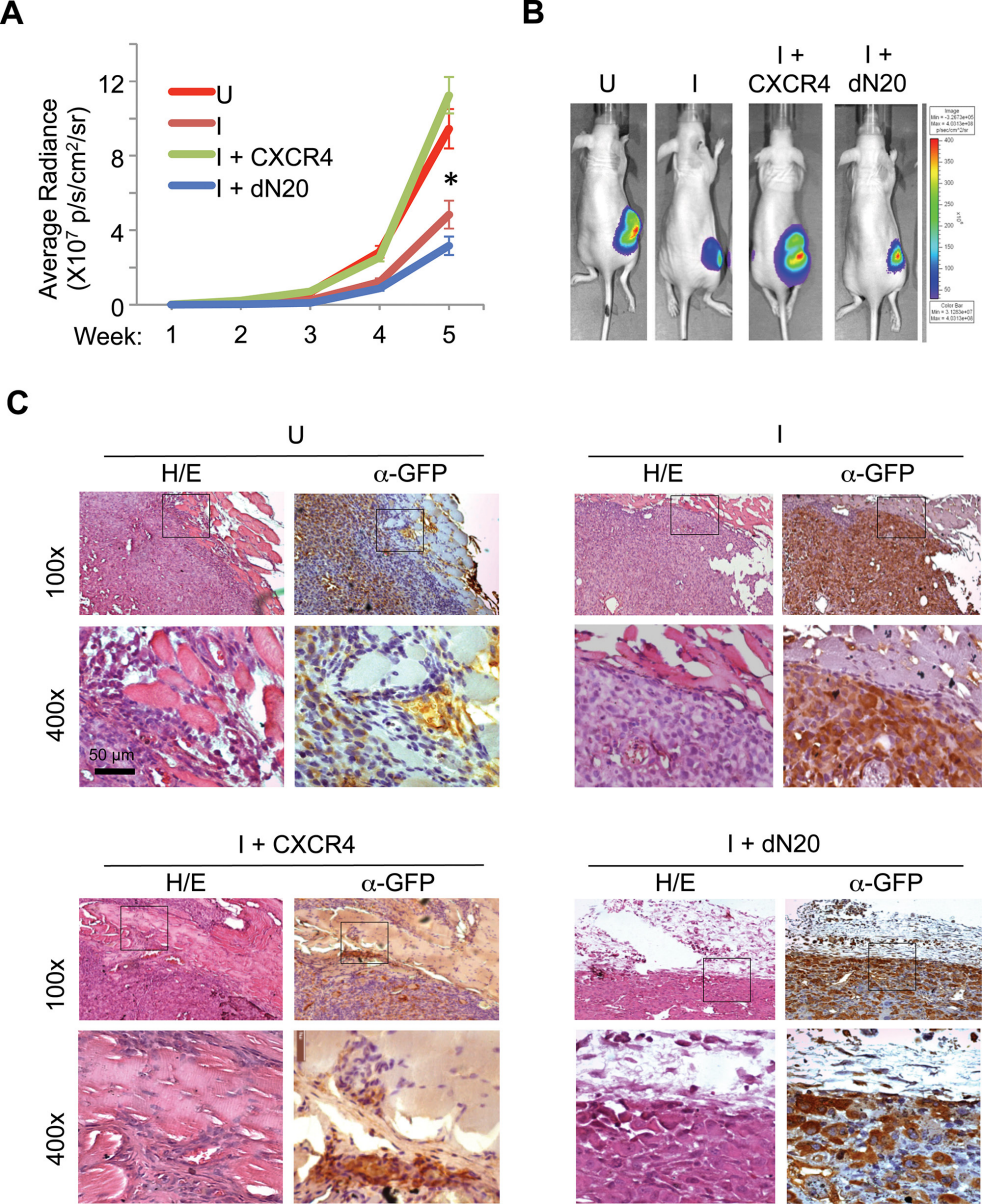
KLF8 activation of CXCR4/CXCL12 signaling is required for invasive growth of the orthotopic breast tumor (**A**, **B**) Overexpression of CXCR4 but not the dN20 mutant prevents the mammary tumor growth from inhibition by KLF8 knockdown. The 231-K8ikd, 231-K8ikd/CXCR4 and 231-K8ikd/dN20 cells were injected into the mammary fat pad of the mice. The mice were fed with Dox-diet (I) or control diet (U). The tumor growth rate was followed up for 5 weeks by BLI analysis (a). *P < 0.05 between the U and I groups and between the I + CXCR4 and I + dN20 groups. Representative BLI images in the end of week 5 are shown (b). (**C**) Overexpression of CXCR4 but not the dN20 mutant prevents the tumor invasion from inhibition by KLF8 knockdown. The above-described tumors were processed for H/E and IHC staining for GFP expression.

To determine the extent to which CXCR4 contributes to primary breast tumor progression downstream of KLF8, we injected the 231-K8ikd cells, the 231-K8ikd cells with stable overexpression of CXCR4 or its dN20 mutant into the mammary fat pad, induced the knockdown of KLF8 *in vivo* and examined the growth and invasion of the orthotopic tumor. The 231-K8ikd cell line stably expresses a GFP-luciferase fusion protein for tracking the tumors by live bio-imaging [16]. As expected, knockdown of KLF8 (I) significantly slowed down the tumor growth (Figure 6A and 6B, compare I with U). However, this reduction was completely prevented by overexpression of CXCR4, but not its dN20 mutant (Figure 6A and 6B, compare I+CXCR4 or I+dN20 with I). Histological analyses revealed that the dramatic inhibition of the tumor invasion into the surrounding tissues by knockdown of KLF8 (Figure 6C, compare I with U) was also well prevented by overexpression of CXCR4, but not its dN20 mutant (Figure 6C, compare I+CXCR4 or I+dN20 with I).

These results suggest that CXCR4 plays a critical role downstream of KLF8 in mediating the primary tumor growth and invasion where interaction with CXCL12 is essential.

### KLF8 promotes CXCR4-dependent lung metastasis

**Figure 7:**
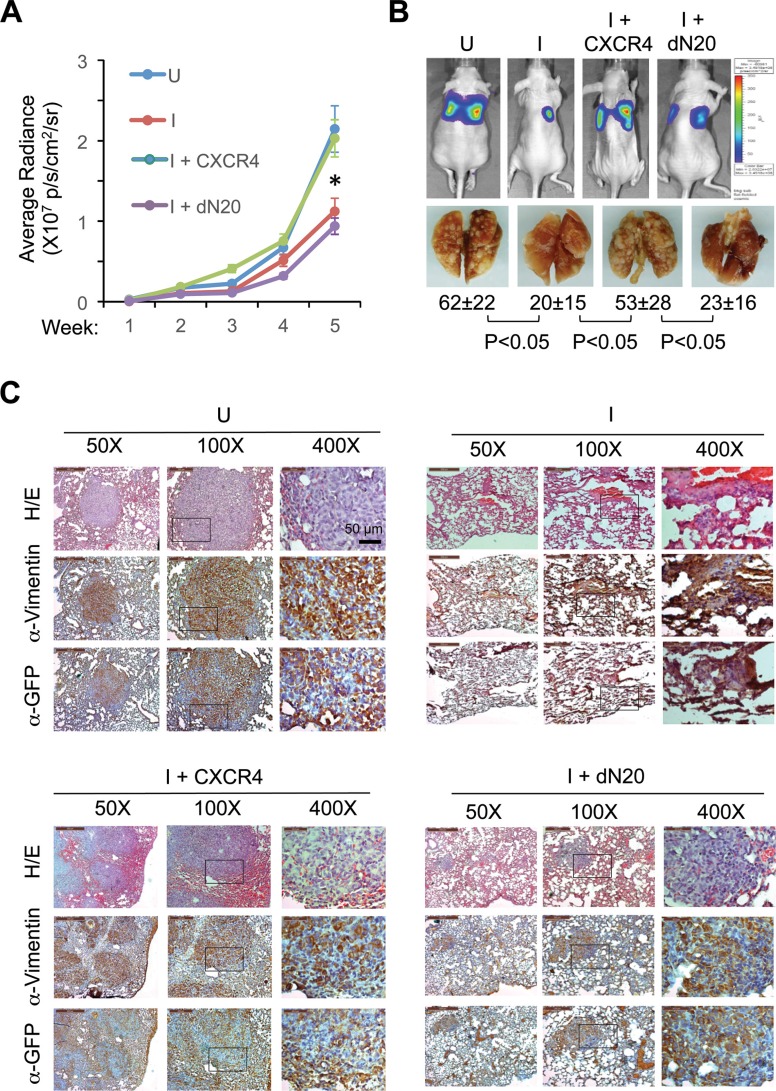
KLF8 activation of CXCR4/CXCL12 signaling is required for lung metastasis (**A**, **B**) Overexpression of CXCR4 but not the dN20 mutant prevents lung metastasis from inhibition by KLF8 knockdown. The 231-K8ikd, 231-K8ikd/CXCR4 and 231-K8ikd/dN20 cells were injected into the tail vein of the mice. The mice were fed with Dox-diet (I) or control diet (U). The lung metastatic rate was followed up for 5 weeks by BLI analysis (a). *P < 0.05 between the U and I groups and between I + CXCR4 and I + dN20 groups. Representative BLI images, metastatic nodules on the whole mount lungs and statistic values in the end of week 5 are shown (B). (**C**) Histological evidence supporting the role of CXCR4 downstream of KLF8 for lung metastasis. The above-described lungs were processed for H/E and IHC staining with antibodies against GFP and human vimentin.

We then examined whether CXCR4 is needed downstream of KLF8 for metastasis. We injected the above-described 231-K8ikd cell lines into the tail veins, induced the knockdown of KLF8 *in vivo* and examined their lung metastasis. Knockdown of KLF8 caused a dramatic decrease in the lung metastatic rate as determined by bioluminescent imaging (BLI) and whole mount lung observation (Figure 7A and 7B, compare I with U). This decrease was again well prevented by overexpression of CXCR4, but not its dN20 mutant (Figure 7A and 7B, compare I+CXCR4 or I+dN20 with I). These results were subsequently verified by histological analyses using hematoxylin and eosin (H/E) staining and immunohistochemical (IHC) staining for the human tumor cell-specific expression of GFP and vimentin (Figure 7C).

Taken together, our results support a critical role of CXCR4 engagement by CXCL12 downstream of KLF8 for breast cancer metastasis.

## DISCUSSION

**Figure 8:**
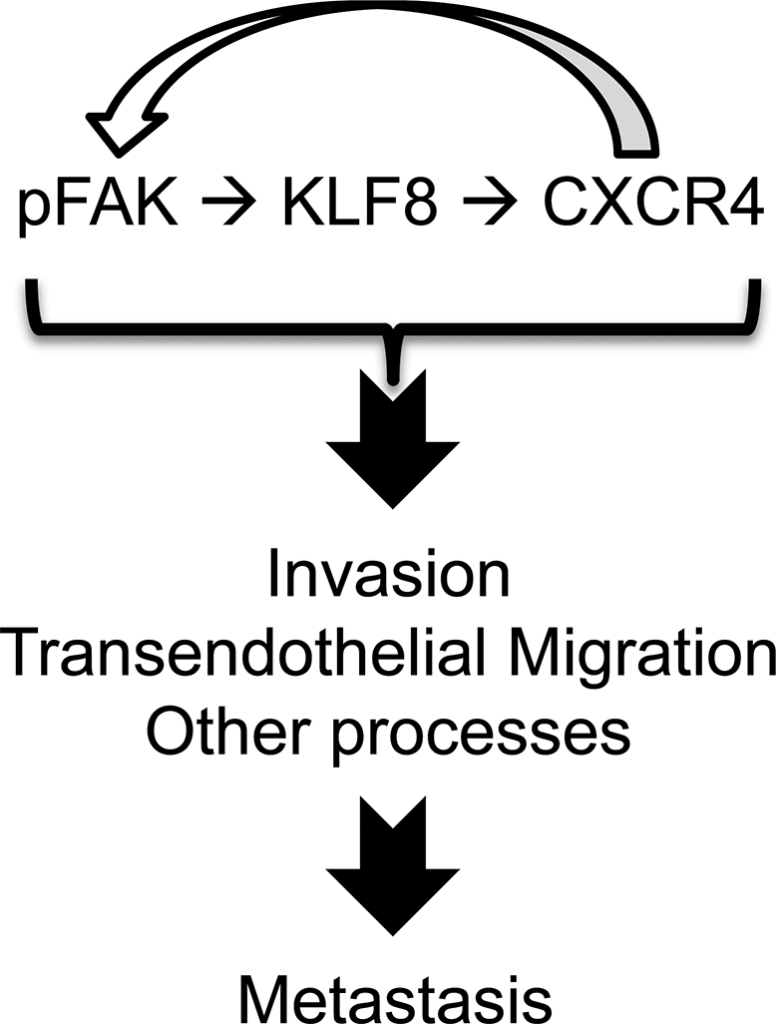
A model of role of the feed-forward signaling loop for metastasis

This study identified CXCR4 as a novel direct target of transcriptional activation by KLF8 and a key mediator of KLF8's role in promoting CXCL12-dependant beast cancer cell migration and invasion required for the invasive growth of the primary tumor as well as TEM essential for the lung metastasis involving a feed-forward activation of FAK (Figure 8).

As shown in Figure 2, KLF8 directly interacts with the CXCR4p GT-box to activate the promoter. Mutation of this GT-box completely abolishes the activation. This result strongly suggests that the GT-box 1 site plays an indispensable role for the promoter activation by KLF8. We noticed that promoter deletion upstream of this GT-box also slightly reduces the promoter activation by KLF8 (Figure 2C), suggesting that those deleted regions, particularly that between the −1230 bp and −1372 bp and that between the −1520 bp and −2088 bp, also play a role in mediating the promoter activation by KLF8. This mode of activation is likely indirect through unknown KLF8 target transcription factors given that KLF8 binds the promoter at the GT-box 1 site (Figure 2E and 2F) and mutation of the other GT-boxes individually did not change the promoter responsiveness to KLF8 (Figure 2D). Several transcription factors have been implicated in activation of the CXCR4 gene promoter [27]. It will be interesting to test if they are regulated by KLF8 and mediate the promoter activation by KLF8 in the cells.

Consistent with our results, it was reported that FAK was activated by CXCL12 stimulation in hematopoietic precursor cells [57, 58], which enhances cell migration towards CXCL12 [57, 59]. In those cells, it appears that activation of PI-3 kinase by Ga_i_ is important in mediating activation of FAK in response to engagement of CXCR4 by CXCL12, although exactly how the activation of PI-3 kinase leads to the activation of FAK was unclear [57, 58]. The study also showed that Src activity is required for the activation of FAK downstream of CXCR4 [58]. We do not know how FAK is activated by the feed-forward loop of KLF8/CXCR4 in response to CXCL12 stimulation in the breast cancer cells used in this study. It is possible that the breast cancer cells use the same mechanisms involving direct activation of PI-3 kinase and/or Src by Ga_i_ as described above. Alternatively, the G proteins could transactivate other receptor proteins such as integrins or EGFR [60] resulting in subsequent downstream activation of FAK [55]. We have demonstrated that both PI-3 kinase and Src play a role in FAK-induced upregulation of KLF8 in fibroblasts and ovarian cancer cells and in the latter activation of SP1 downstream of Akt plays a likely role [7, 8]. Whether FAK upregulates KLF8 in the breast cancer cells by the same mechanism remains to be determined. In addition, it would also be interesting to investigate whether this feed-forward signaling loop can self-stand. In our 10A-iK8 cells, this feed-forward loop is initiated by HA-KLF8 expression in combination with CXCR4 engagement that is achieved by including both doxycycline and CXCL12 in the medium. It is possible that, once initiated, the loop can self-stand even if the ectopic KLF8 expression is subsequently turned off. However, the feed-forward loop would not last unless the CXCL12 ligand is constitutively available for the receptor engagement. In view of this, on a physiological level the cell may attenuate the loop primarily in response to diminished extracellular CXCL12 expression and/or through rapid receptor internalization. Nevertheless, it will be important to determine the detailed signaling mechanisms that operate the critical feed-forward wheel of KLF8/CXCR4/FAK in breast cancer cells.

This is the first demonstration of the role for KLF8 in promoting TEM requiring CXCR4 engagement by CXCL12. For such TEM to occur, the cancer cell must be able to make a local trip along a CXCL12 gradient to approach the vascular vessels. This trip requires the cancer cell to be able to leave the primary tumor, migrate and degrade or re-model the extracellular matrix while invading through the stroma and the availability of the local gradient of CXCL12 and vascular vessels. KLF8 is well known to promote cell dissociation and migration by inducing EMT [6, 11–13] and invasion by upregulating MMP expression and activity [16, 19]. FAK is a potent migration-promoting protein upstream of various signaling effector proteins such as p130Cas and Crk [55, 59], which was also shown in CXCL12-stimulated cells [57, 58]. Given the novel feed-forward signaling wheel identified (Figures 5 and 8), it is likely that CXCR4 engagement by CXCL12 has an important impact on the regulation of the effector proteins downstream of both KLF8 and FAK, investigation of which is in progress. Cell movement associated with migration, invasion and TEM requires cytoskeletal rearrangement such as the formation and dynamic change of filapodia and lamellipodia. It will be interesting to determine whether the feed-forward signaling loop serves as an important molecular/signaling mechanism underlying this critical cellular change in metastatic cancer cells. Tissue damaging conditions such as hypoxia, toxins and ionized irradiation induce a CXCL12-rich environment for recruiting CXCR4^+^ stem cells and leukocytes to the site that requires tissue repair or regeneration [26, 36, 46]. Indeed, vascular endothelial cells under these stress conditions produce increased level of CXCL12 [61]. In addition, the high level of CXCL12 secreted by tumor-associated stromal cells upon hypoxia is critical for tumor angiogenesis [28]. It is thus important to investigate if KLF8 plays a role in tumor angiogenesis and resistance to anti-cancer therapies such as radiotherapy and if CXCR4 is involved.

We demonstrate that KLF8 plays a critical role in both invasive growth of the local primary tumor (Figure 6) and the lung metastasis (Figure 7) in a CXCR4-dependent manner. It has been reported that cancer-associated fibroblasts can release CXCL12 at the primary tumor site to promote the primary tumor growth and invasion [28]. In view of this, the feed-forward signaling loop may also be a key in promoting primary tumor outgrowth. In addition to EMT, invasion and potential tumor angiogenesis discussed above, the remarkable impact of the feed-forward signaling on TEM indicates that KLF8 may be critical in promoting intravasation and/or extravasation via CXCR4-dependent TEM during metastasis. On reaching the foreign tissue, successful metastasis requires the tumor cells to respond to chemotactic signals in order to survive and ultimately colonize. Interestingly, FAK has recently been implicated in promoting the breast cancer colonization in the lungs [62]. It will be interesting to test if KLF8 also participates in regulating this final rate-limiting metastatic step and whether CXCR4 and CXCL12 are needed. The expression of CXCL12 has been found to be highest in the tissues of the lungs, liver, bone marrow and brain as well as lymph nodes [34, 37]. Indeed, these tissues tend to be the metastatic sites of CXCR4-high cancer cells [34, 38, 53, 63–65]. It will be worth determining if KLF8-high cancer cells tend to colonize in these other CXCL12-high tissues in addition to the lungs.

This study opens a new opportunity for understanding molecular mechanisms critical for targeted therapeutic control of breast cancer. Given the key role for both FAK and CXCR4 in breast cancer metastasis, FAK- and CXCR4-targeted therapies have been explored in pre-clinical tests and clinical trials [34, 48, 66–69]. However, major obstacles remain due mainly to unbearable side effects on normal tissues. The primary cause is the wide, if not ubiquitous, expression and physiological function of these two proteins. Indeed, knockout of either FAK or CXCR4 is embryonically lethal [70, 71]. Thus, it is imperative to identify the mechanism by which the expression and/or activation of FAK and CXCR4 is induced specifically in tumor cells. Importantly, the expression of KLF8 in normal tissues is much lower than that in cancer tissues [6] and knockout of KLF8 is not embryonically lethal [72]. Thus, the identification of KLF8 as a critical feed-forward inducer of CXCR4 expression and activation of FAK in breast cancer cells presents an excellent therapeutic target to correct aberrant signaling of both FAK and CXCR4 specifically in cancer cells. Together with our previous reports demonstrating a critical implication of KLF8 in malignant progression of breast cancer [6, 8, 10, 11, 15–18], this study further underscores KLF8 as an important regulator of breast cancer pathogenesis and malignancy and a potential therapeutic target with less side effects.

## MATERIALS AND METHODS

### Antibodies and reagents

Antibodies include mouse monoclonal for HA-probe (F-7) (sc-7392), β-actin (C4) (sc-47778), and GFP (sc-101525) and rabbit polyclonal for FAK (sc-557) (Santa Cruz Biotechnology Inc, Dallas, TX, USA), CXCR4 rabbit polyclonal Ab (Ab-2074) (Abcam, Cambridge, MA, USA), and pY397-FAK rabbit monoclonal Ab (Invitrogen, 44625G, Carlsbad, CA, USA), and vimentin mouse monoclonal Ab (550513) (BD Pharmingen, San Jose, CA, USA). Anti-KLF8 rabbit polyclonal Ab was described previously [18, 19]. Peroxidase substrate kit (DAB) (SK-4100) was purchased from Vector laboratories Inc. (Burlingame, CA, USA). The CXCR4 inhibitor AMD3100 (or octahydrochloride hydrate) (A5602) and the FAK inhibitor PF573228 (PZ0117) were purchased from Sigma (St. Louis, MO, USA). Recombinant human CXCL12 (300–28A) was purchased from Peprotech (Rocky Hill, CT, USA).

### Plasmid construction, cell line generation, cell culture and transfection

The mammalian expression plasmids pKH3, pKH3-KLF8 and pKH3-mKLF8 were previously described [16, 21]. The human CXCR4 gene promoter (−2088 base pairs) was cloned by PCR from template genomic DNA isolated from MDA-MB-231 cells using the Wizard Genomic DNA purification Kit (A1120) from Promega (Madison, WI, USA). The PCR product was cut with Kpn1 and Nhe 1 and ligated to the respective sites in the pGL3basic vector (Promega, Madison, WI, USA) to form the pGL3b-CXCR4p promoter reporter plasmid. The promoter truncation mutant plasmids were constructed by PCR deletion of desired promoter fragment from the pGL3b-CXCR4p plasmid. The GT-box specific mutations were created by site-directed mutagenesis overlapping PCR [73–75] using the pGL3b-CXCR4p vector as the template. The CXCR4 cDNA was PCR-synthesized from mRNA isolated from MDA-MB-231 cells and cloned into the pKH3 vector between the SmaI and EcoRI sites to form the pKH3-CXCR4 plasmid. The amino terminal 20 amino acids of CXCR4 in the plasmid were removed by deletion PCR to obtain the dominant-negative mutant plasmid pKH3-CXCR4-dN20. To construct lentiviral vectors pLVZP-CXCR4 and pLVZP-CXCR4-dN20, we sub-cloned HA-CXCR4 and HA-CXCR4-dN20 from the pKH3-CXCR4 and pKH3-CXCR4-dN20 plasmids respectively, into the lentiviral vector pLVPZ between the PstI and NotI sites [19] by PCR. All the primers are listed in Supplementary Information. All the constructs were verified by DNA sequencing. The 231-K8ikd cell line expressing ectopic CXCR4 or its dN20 mutant was generated by infecting the 231-K8ikd cells with the lentivirus of pLVZP-CXCR4 or pLVZP-CXCR4-dN20 followed by puromycin selection. The HEK293, NIH3T3, MCF-10A, MDA-MB-231, the MCF-10A that expresses doxycycline-inducible KLF8 (10A-iK8), the MDA-MB-231 that expresses doxycycline-inducible shRNA against KLF8 (231-K8ikd) were described previously [16]. These cells also stably express a GFP-luciferase fusion protein for live bio-imaging tracking of the cells *in vitro* and *in vivo*. These cells were maintained in Dulbecco's modified Eagle's medium/F-12 with 5% horse serum or Dulbecco's modified Eagle's medium with 10% fetal bovine serum. The inducible cell lines were maintained under uninduced (U, in the absence of doxycycline) or induced (I, in the presence of doxycycline) conditions depending on the experimental need. Doxycycline hydrochloride was purchased from Sigma (D3072) (St. Louis, MO, USA). Plasmid DNA transfections were performed using Lipofectamine 2000 (Invitrogen, Grand Island, NY, USA).

### Quantitative real time-PCR (qRT-PCR) and western blotting

Cell lines and antibodies used have been described above. qRT-PCR was performed as previously described [9–11, 16, 18] (See Supplementary Information for primers). Western blotting for CXCR4 was conducted as previously described by A. Marchese [76, 77].

### Promoter reporter, chromatin immunoprecipitation (ChIP) and biotinylated oligonucleotide precipitation (BOP) assays

These assays were performed essentially as previously described [16, 18]. For reporter assays, cells were co-transfected with each of the pGL3b-CXCR4p reporter or mutant plasmids and pKH3-KLF8, pKH3-mKLF8 or pKH3 vector alongside a Renilla luciferase reporter internal control for 16–18 hours followed by quantification of luciferase activity. ChIP assays were performed by transfecting HEK293 cells with pKH3-KLF8 or pKH3 control vector followed by anti-HA co-precipitation of promoter fragments. HA antibody-conjugated agarose beads were purchased from Sigma (St. Louis, MO, USA). The control IgG was purchased from Jackson ImmunoResearch, Inc. (West Grove, PA, USA). For the BOP assays, the lysates of HEK293 cells transfected with pKH3-KLF8 were processed for analysis. (See Supplementary Information for ChIP primers and BOP oligonucleotides).

### Boyden chamber migration and matrigel invasion assays

The chambers were purchased from BD Biosciences (San Jose, CA, USA) and the assays were performed as instructed by the manufacturer with necessary modifications as previously described [11, 16–19]. Cells were incubated inside the upper inserts in serum-free conditions. In some specified cases, the cells were pre-treated with AMD3100 (35 ng/ml) or PF573228 (1 μM) for three hours prior to seeding into the insert. After allowed for 16–20 hours, duration shorter than population doubling times of the cells to migrate or invade towards recombinant CXCL12 (200 ng/ml) contained in serum free medium in the bottom chamber, the cells on the undersurface of the insert were stained with 0.2% Crystal Violet in 10% methanol and processed for quantitative analysis.

### Transendothelial migration (TEM) assay

TEM assay was performed using the CytoSelect Tumor TEM assay kit (CBA-216, Cell Biolabs, Inc. San Diego, CA, USA). Briefly, HUVEC cells (25,000–50,000) (CRL-2922, ATCC, Manassas, VA, USA) were allowed up to 72 hours to form a monolayer on the upper surface of the membrane inside the insert and then activated by overnight serum starvation or TNFα treatment. The 10A-iK8 or 231-K8ikd cells (50,000) were incubated with the Cytotracker dye for 1 hour prior to further incubation in the insert on top of the HUVEC monolayer in the presence or absence of doxycycline. Depending upon specific experimental need, AMD3100 (35 ng/ml) was included to inhibit CXCR4 prior to and during the 22-hour assay time. The cells that migrated through the HUVEC monolayer were lysed and quantified using a Fluorescence multi-plate reader (PolarStar Omega, BMG LabTech, Cary, NC, USA).

### Bioluminescent imaging (BLI) analysis of tumor growth and metastasis

Female Balb/c nude mice (NCI, Frederick, MD, USA) of 4–6 weeks old (six per group) were used for testing orthotopic tumor growth and lung metastasis by the 231-K8ikd cells with or without the stable overexpression of CXCR4 or its dN20 mutant. The cells were implanted prior to induction of KLF8 knockdown in the mice. The mammary fat pad and tail vein injection of the cells, the Dox Diet feeding, and the weekly BLI monitoring of the tumor growth or lung metastasis were performed as previously described [16–19]. The mice were housed and maintained in specific pathogen-free conditions in the UCF vivarium approved by the American Association for Accreditation of Laboratory Animal Care and in accordance with current regulations and standards of the United States Department of Agriculture, United States Department of Health and Human Services, and the National Institute of Health. The animal experiments were guided by the university-approved IACUC protocol with thorough consideration of humane care of the mice.

### Hematoxylin and eosin (H/E) and immunohistochemical (IHC) staining

The collection and preparation of the primary and metastatic tumor tissues, the human breast cancer tissue array used, and the histological analysis procedures were previously described [9, 16–19]. The antibodies for KLF8, CXCR4, GFP and vimentin were described above.

### Statistical and bioinformatical analysis

Mean +/− standard deviation is presented with a minimum of three observations per group. Student's *t*-test, unpaired, paired or single sample, with the Bonferroni correction for the multiple comparisons was applied as appropriate. The two by two tables were analyzed by Fisher's exact test. The alpha level of 0.05 was used to determine difference with statistical significance. Breast cancer microarray data of GEO DataSet (GSE9014) was downloaded from GEO website (http://www.ncbi.nlm.nih.gov/geo/) [78]. RNA sequencing data of 817 breast cancer samples was downloaded from TCGA Data portal (https://tcga-data.nci.nih.gov/tcga/tcgaHome2.jsp) [79]. Pearson's correlation was analyzed between KLF8 and CXCR4.

## SUPPLEMENTARY MATERIALS


