# Supplementary material for: Krüppel-like factor 8 activates the transcription of C-X-C cytokine receptor type 4 to promote breast cancer cell invasion, transendothelial migration and metastasis
Source: Oncotarget. 2016 Mar 14;7(17):23552–68. doi: 10.18632/oncotarget.8083 (PMC5029647; doi:10.18632/oncotarget.8083)
Supplement: Supplementary file 1 [file oncotarget-07-23552-s001.pdf]

Krüppel-like factor 8 activates the transcription of C-X-C cytokine receptor type 4 to promote breast cancer cell invasion, transendothelial migration and metastasis

Supplementary Materials

The PCR primers used to clone the human CXCR4 gene promoter:

CXCR4p-F, 5′- ACG CGG TAC CGT GCA GCT TAC GGT C -3′

CXCR4p-R, 5′- TGT CCG CTA GCA TGG TAA CCG CTG GTT C-3′.

Primers used to generate the truncation mutants of the human CXCR4 gene promoter:

The CXCR4p-R primer described above was used for constructing all the truncations. All the forward primers listed below contained a Kpnl restriction site.

-1520CXCR4p-F, 5′-AAC TGG TAC CGG AAC ACT CCA GAC TG -3′

-1372CXCR4p-F, 5′ - TCG GTG GTA CCT ATC TCC GCA GCG-3′

-1230CXCR4p-F, 5′-TGC AAG GTA CCG CCA GTC TGA GAT CG -3′

-397CXCR4p-F, 5′- ACG CGG TAC CGA GAG ACG CGT TCC -3′

-217CXCR4p-F, 5′- ACC TGG TAC CTC CTT CCT CGC GTC TGC - 3′

-150CXCR4p-F, 5′- ATG CGG TAC CCT CGG AGC GTG T - 3′

Primers used to construct the GT-box specific mutants of the human CXCR4 gene promoter:

CXCR4p-mGT1-F, 5′- TCC CCT CTC ACG CCT TCT CCC TC -3′

CXCR4p-mGT1-R, 5′- AAG GCG TGA GAG GGG AGA GGG GCA -3′

CXCR4p-mGT4-F, 5′- CGC CCT CTC AAG TCT CCG CGC CGG AG - 3′

CXCR4p-mGT4-R, 5′- AGA CTT GAG AGG GCG GGG GAG CGA GTG -3′

CXCR4p-mGT5-F, 5′- AGC GGC TCT CAC GCC TGC GGA C- 3′

CXCR4p-mGT5-R, 5′- AGG CGT GAG AGC CGC TGC GGA GAT A- 3′

CXCR4p-mGT7-F, 5′- AGC CAC TCT CAT CCA AGA ACC TGG- 3′

CXCR4p-mGT7-R, 5′ - TTG GAT GAG AGT GGC TGG AAG AGG- 3′

These mutant primer sets for each of the mutant GT-box (mGT) were used along with the CXCR4p-F/CXCR4p-R primer set described above.

Primers used to clone the cDNA of the human CXCR4 and the dN20 mutant:

hCXCR4-Sma-F, 5′- TCC CCC GGG CGT GGA GGG GAT CAG TAT ATA -3′

hCXCR4-dN20-Sma-F, 5′- TCC CCC GGG CTA TGA CTC CAT GAA GGA AC -3′

hCXCR4-RI-R, 5′- AGG AAT TCT TAG CTG GAG TGA AAA CTT G -3′

Primers used to construct the pLVZP-CXCR4 and pLVZP-CXCR4-dN20:

pKH3-F, 5′- CCC AAG CTT CTG CAG GTC G - 3′

pLVPZ-CXCR4-Not1-R, 5′ - ATA AGA ATG CGG CCG CTT AGC GAA AAC -3′

Primers used for qRT-PCR:

hCXCR4-RT-F, 5′ - GGC GTC TGG ATC CCT GCC CT -3′

hCXCR4-RT-R, 5′- TGC GCT TCT GGT GGC CCT TG -3′

hKLF8-F, 5′-TCT GCA GGG ACT ACA GCA AG -3′

hKLF8-R 5′-TCA CAT TGG TGA ATC CGT CT -3′

hGAPDH-F, 5′- TCG TAC GTG GAA GGA CTC A -3′

hGAPDH-R, 5′- CCA GTA GAG GCA GGG ATG AT -3′

The ChIP assay primers directed along the nucleotide sequence flanking the GT1 box of the CXCR4 promoter:

CXCR4p-GT1 -ChlP-F, 5′ - TTC CCA AGC CGC GCA CCT CTC -3′

CXCR4p-GT1 -ChlP-R, 5′- ATG CGG TGG CTA CTG GAG CAC TC -3′

The BOP assay oligonucleotides spanning the GT1 site in the human CXCR4 promoter:

WT-sense: 5′-CCC CTG CGC CCT CCT TCC TCG CGT CTG CCC CTC TCC CCC ACC CCG CCT TCT CCC TCC CCG CCC CAG CGG CGC ATG CGC CGC GCT CG-3′

WT-antisense: 5′-CGA GCG CGG CGC ATG CGC CGC TGG GGC GGG GAG GGA GAA GGC GGG GTG GGG GAG AGG GGC AGA CGC GAG GAA GGA GGG CGC AGG GG-3′

mGTl-sense: 5′-CCC CTG CGC CCT CCT TCC TCG CGT CTG CCC CTC TCC CCA AAC ACG CCT TCT CCC TCC CCG CCC CAG CGG CGC ATG CGC CGC GCT CG-3′

mGTl-antisense: 5′-CGA GCG CGG CGC ATG CGC CGC TGG GGC GGG GAG GGA GAA GGC GTG TTT GGG GAG AGG GGC AGA CGC GAG GAA GGA GGG CGC AGG GG-3′.
